# Design and Research on Working Teeth of Separable-Tooth Cathode for Electrochemical Machining

**DOI:** 10.3390/mi17101155

**Published:** 2026-09-30

**Authors:** Bo Li, Qingliang Li, Lingfei Kong, Tianqi Cao

**Affiliations:** 1 School of Mechanical Engineering, Xi’an University of Technology, Xi’an 710048, China18560159060@163.com (T.C.); 2 Engineering Training Center, Xi’an Polytechnic University, Xi’an 710600, China; 3 Xi’an Kunlun Industry Group Co., Ltd., Xi’an 710043, China; liqq20072008@126.com

**Keywords:** electrochemical machining (ECM), separable-tooth cathode, variable-pitch helical structure, inter-electrode gap, machining accuracy

## Abstract

In the electrochemical machining (ECM) of variable-pitch internal helical grooves, a conventional integral cathode is subject to a kinematic constraint: the working teeth are rigidly fixed to the cathode body and therefore cannot follow a helix angle that varies continuously along the axis, and the resulting angular mismatch widens the groove and degrades the lands. To address these critical issues, this paper proposes a novel separable-tooth cathode design. As the cathode feeds along the variable-pitch helical path, each toothed disk is independently rotated to the corresponding angle by a built-in micro-motor embedded within the cathode body. This adaptive rotation ensures that the envelope of the working teeth remains consistently aligned with the real-time helix angle, thereby enabling the precise forming of variable-pitch helical features. Furthermore, inspired by the biomimetic reduction mechanism, four groups of bionic surface structures with different geometric parameters are designed and fabricated on the working teeth. Numerical simulations are conducted using COMSOL Multiphysics to investigate the correlations among the bionic structural parameters, electrolyte flow velocity, turbulent characteristics, and wall shear rate. The results indicate that the optimized bionic morphology with a spacing s = 0.2 mm and a height h = 0.05 mm significantly reduces the near-wall frictional resistance and effectively stabilizes the flow field distribution in the inter-electrode gap. Finally, systematic ECM verification experiments are performed using the proposed split-type bionic cathode. A metrological analysis of the sectioned workpiece reveals that the machined inner hole achieves a surface roughness of S_a_1.6 μm, a roundness error of 0.02 mm, and a dimensional consistency within 0.02 mm. The experimental results demonstrate that the developed separable-tooth cathode satisfies the stringent design specifications, simultaneously guaranteeing a high machining accuracy and efficient material removal in the precision ECM of complex helical components.

## 1. Introduction

Electrochemical machining (ECM) has become an irreplaceable processing method for difficult-to-machine materials and complex structural components, especially for internal helical structures with high precision requirements. Traditional ECM cathodes usually adopt an integrated cylindrical structure, which is generally prepared by pressing a copper body and epoxy resin under a certain pressure. During machining, the cathode teeth move axially and feed according to the given helical equation [1,2]. For fixed-pitch helical structures, since the helix angle remains unchanged, the cathode can maintain good matching with the machined profile, thereby ensuring the dimensional accuracy and surface quality of the internal grooves.

**Figure 1:**
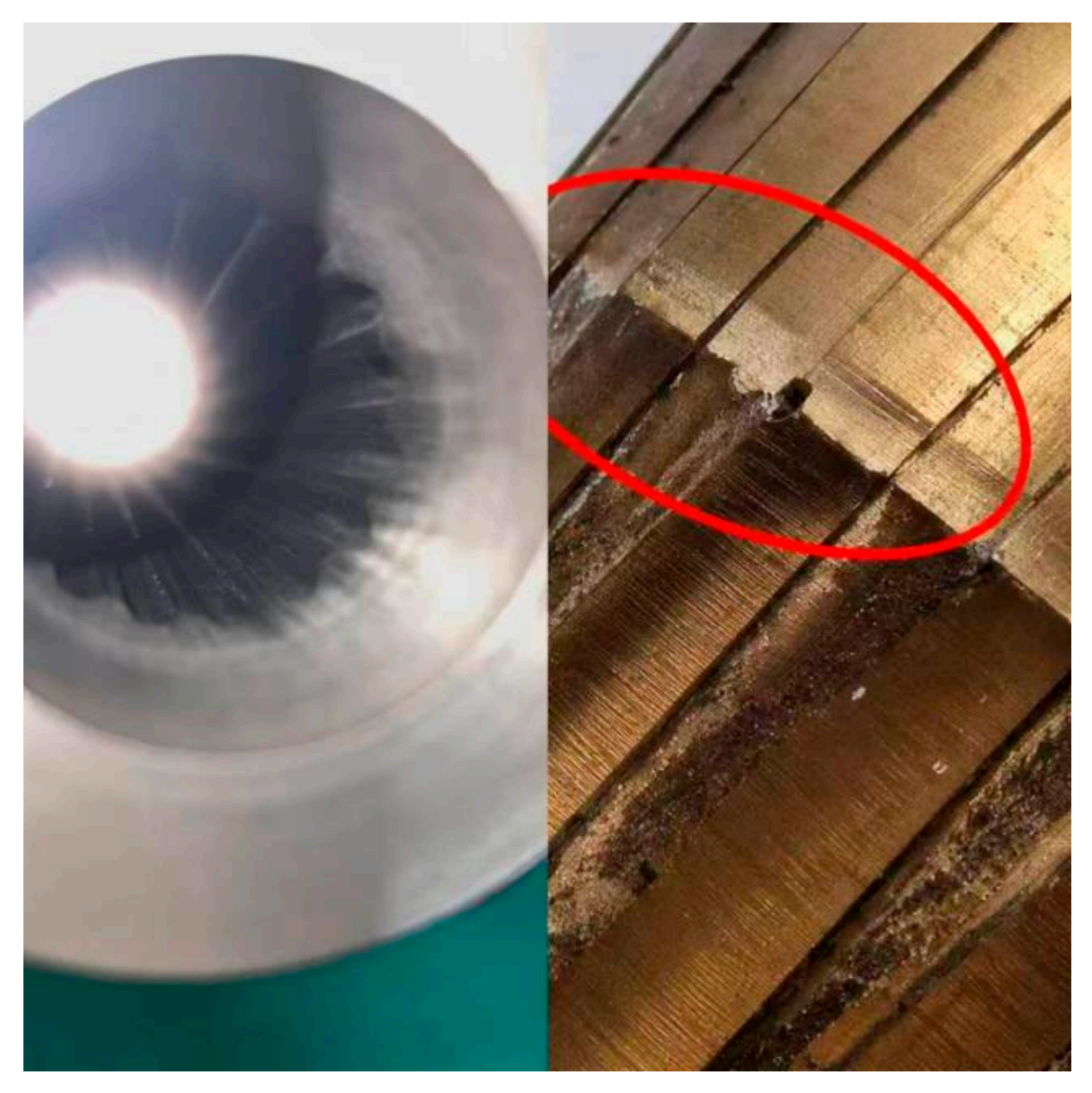
Cathode burn and sagging pits.

However, for variable-pitch helical structures, the helix angle changes continuously along the axial direction. Under such conditions, the rigid teeth of the integral cathode cannot adjust their posture adaptively. As illustrated in Figure 1, this mismatch inevitably leads to severe defects such as excessive groove widening, local collapse pits, deepened groove bottoms, and even cathode burning. To alleviate these problems, researchers have proposed improved cathodes with wedge-shaped teeth and adopted auxiliary strategies including computer numerical control, real-time power regulation, and electrolyte parameter optimization. Tang et al. carried out electrolyte-formulation experiments based on orthogonal and grey-relational analysis, together with the cathode structural design and simulation of the cathode teeth, and machined constant-twist helical components on a purpose-built horizontal CNC electrochemical machine [3]; their subsequent work likewise addresses structural and process-parameter optimization under constant-lead, fixed-cathode conditions. Shen and Fan proposed a combined cathode in which ten toothed disks are inner-meshed with gears of different modules and tooth numbers and are driven by a micro-motor embedded in the cathode body, so that each disk is rotated to a different angle and the working teeth remain aligned with the rifling twist [4]. This paper presents an articulated ECM cathode whose working teeth are constituted by 30 independently rotatable toothed disks, each driven through a transmission integrated in the cathode body so that its angular position tracks the local helix angle of the target groove as the tool advances axially. Nevertheless, due to the discontinuous distribution of separated teeth, steps are easily formed between adjacent tooth units during machining, which induce severe cavitation, flow separation, and vortices in the inter-electrode gap. These flow disorders further deteriorate the flow field stability and reduce the final forming accuracy. Thus, it is urgent to carry out flow field optimization in the inter-electrode gap to improve the processing stability of the Separable cathode.

Biomimetic drag reduction technology provides an effective solution to the above flow field problems. Inspired by the surface morphology of shark skin, NASA successfully applied bionic micro-riblets to aerospace vehicles to optimize turbulent boundary layers and reduce flow resistance [5]. Belcher et al. systematically studied four typical bionic structures, including V-shaped, U-shaped, L-shaped, and spaced V-shaped riblets. Their experimental results confirmed that the V-groove riblet structure exhibits the best drag reduction effect, with a maximum drag reduction rate of approximately 10% [6,7,8]. Finally, a series of ECM experiments are carried out to verify the feasibility of the proposed bionic separable-tooth cathode. The measurement results of machined inner holes show that the novel cathode meets the high-precision manufacturing requirements and effectively improves both machining accuracy and surface quality.

## 2. Separable-Tooth Cathode Design

### 2.1. Overall Design

**Figure 2:**
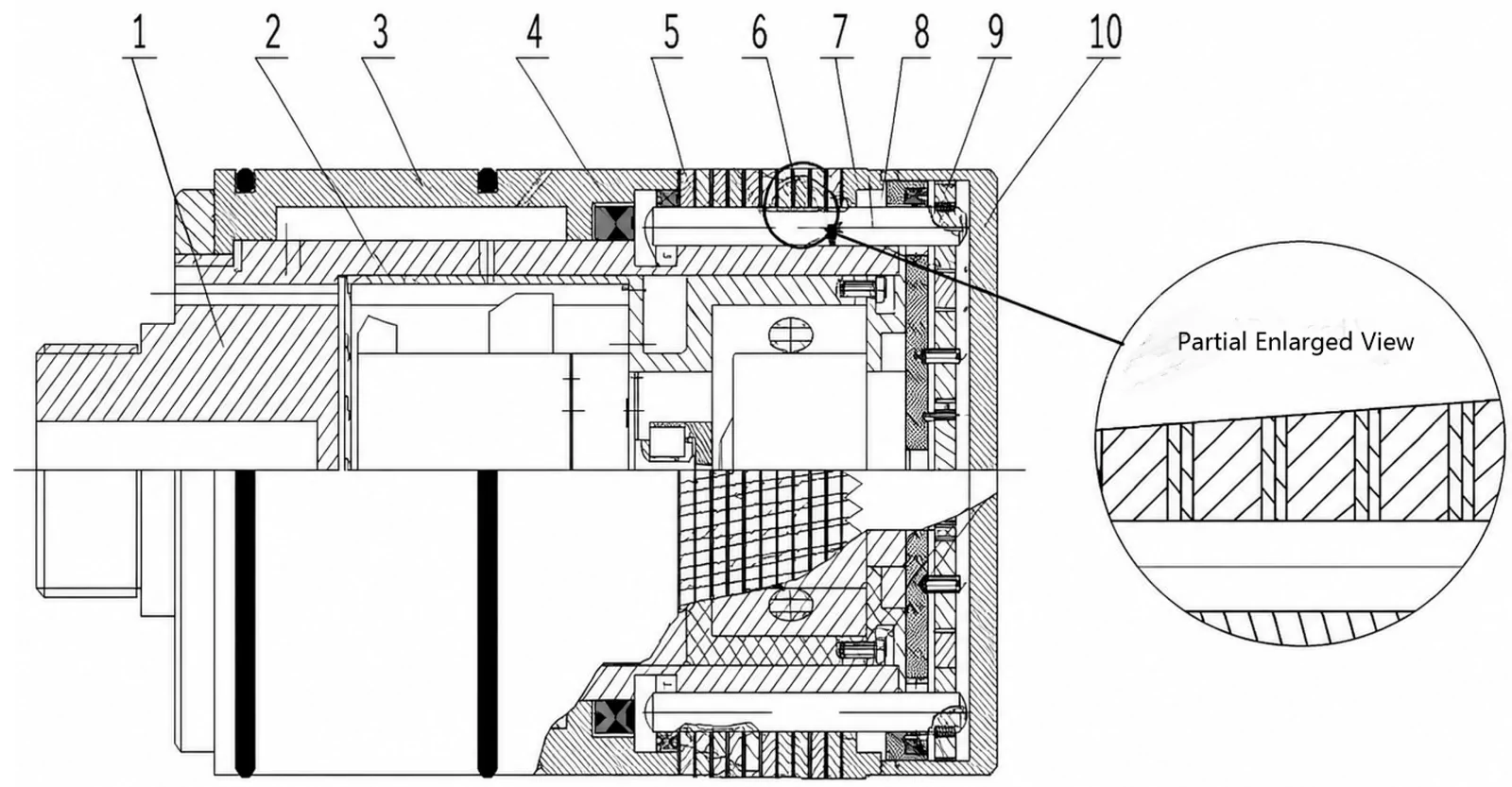
Overall bionic cathode structure and working tooth diagram. Overall bionic cathode structure; Working tooth diagram. 1—cathode body; 2—servo motor; 3—front guide; 4—reducer; 5—working toothed disc; 6—liquid-augmenting disc; 7—drive gear; 8—drive shaft; 9—rear guide; 10—transmission gear.

The overall cathode scheme is shown in Figure 2. The 48 working teeth are composed of 30 gear-shaped working toothed discs with different reference circle diameters and different thicknesses. Each gear-shaped working toothed disc is internally meshed with its corresponding drive gear mounted on the drive shaft. While the cathode moves according to the variable-pitch helix equation, the gear-shaped working toothed discs are driven by the transmission mechanism powered by the micro-motors embedded in the cathode body to rotate relative to the cathode body. Since each gear-shaped working toothed disc has a distinct reference-circle diameter, each disc rotates through a different angle relative to the cathode body. Consequently, the entire working tooth remains aligned with the helix-angle direction of the machined helix along its longitudinal direction.

For a variable-pitch helix whose helix angle starts from 0°, if the working tooth is divided into 10 gear segments with trapezoidal teeth, the envelope dimension of the working tooth is considerably larger than that of the target helix when the helix angle equals 0°. Owing to the excessively large rotation angle, the step height generated by the misalignment can reach 0.7 mm. This step obstructs the normal electrolyte flow and induces severe vortices, flow separation, and cavitation within the flow field, which degrades the machining accuracy. Worse still, short-circuit faults may occur and interrupt the machining process. To solve these problems, a scheme is proposed in which the working tooth is divided into 30 gear segments with variable tooth thickness.

### 2.2. Calculation of Working Tooth Parameters

Like keyway components such as splines and double keys, helical lines are also inner hole grooves. The difference lies in that the cathode working teeth not only undergo axial feed motion but also radial motion. To ensure the forming accuracy, keeping a constant radial inter-electrode gap of 0.5–0.7 mm is conducive to the formation of a uniform flow field, and the inter-electrode gap is selected as Δ = 0.5 mm in this paper. The inter-electrode gap is generally between 0.5 mm and 0.7 mm, and, here, the inter-electrode gap is selected as ∆ = 0.5 mm.

**Figure 3:**
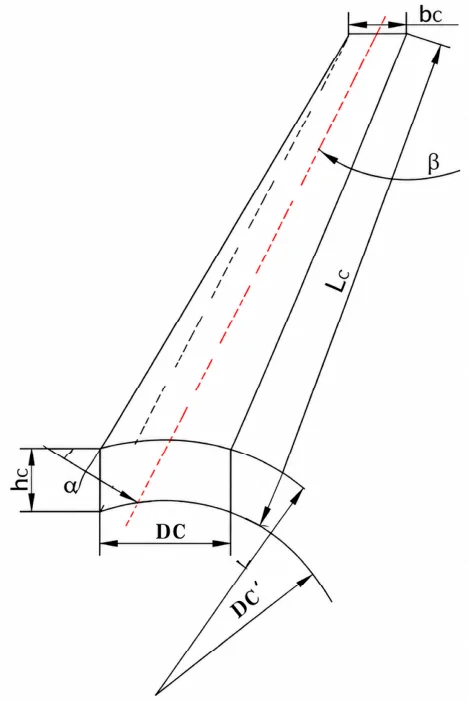
Working tooth parameters.

Set the major diameter (land diameter) *D*_a_ = 160 mm, the minor diameter (groove diameter) *D* = 155.4 mm, and the groove width *B* = 6.3 mm, and the working tooth length is set as 50 mm. The working tooth parameters are shown in Figure 3, and the remaining parameters are calculated as follows:

$$\mathrm{The}\;\mathrm{working}\;\mathrm{tooth}\;\mathrm{large}\text{-}\mathrm{end}\;\mathrm{diameter}\;D_{c}=160-2\times 0.5=159\mathrm{mm};$$

$$\mathrm{The}\;\mathrm{working}\;\mathrm{tooth}\;\mathrm{small}\text{-}\mathrm{end}\;\mathrm{diameter}\;D_{c}=D_{a}-2\times 0.5=154.4\mathrm{mm};$$

$$\mathrm{The}\;\mathrm{working}\;\mathrm{tooth}\;\mathrm{large}\text{-}\mathrm{end}\;\mathrm{width}\;B_{c}=B-2\times 0.5=5.3\mathrm{mm};$$

$$\mathrm{The}\;\mathrm{working}\;\mathrm{tooth}\;\mathrm{large}\text{-}\mathrm{end}\;\mathrm{height}\;h_{c}=\frac{D_{c}-{D\prime }_{c}}{2}=\frac{159-154.4}{2}=2.3\mathrm{mm};$$

$$\tan\beta =\frac{h_{c}-{h\prime }_{c}}{L_{c}};$$

$$\tan\alpha =\frac{B_{c}-b_{c}}{2L_{c}};$$

where:

α—half face angle of the working tooth, (°);

β—half cone angle of the working tooth, (°).

Considering that it is easier to control the dimensional accuracy of the formed helix, let α=β:

The small-end width of the working tooth $b_{c}=B_{c}-2h_{c}+2{h\prime }_{c}$;

Because the small-end height of the working tooth ${h\prime }_{c}=0\;\mathrm{mm}$, therefore, the small-end width of the working tooth

$$b_{c}=B_{c}-2h_{c}=5.3-2\times 2.3=0.7\;\mathrm{mm};$$

The small-end height of the working tooth ${h^{\prime }}_{c}=0\;\mathrm{mm}$;

The working length of the working tooth $L_{c}=50\;\mathrm{mm}$.

### 2.3. Variable-Pitch Helix Kinematics

Let X be the axial coordinate measured from the start of the rifling (mm) and Y the developed length of the rifling (mm). The local helix angle α then satisfies

tan α(X) = dY/dX

The rifling of the present workpiece consists of three segments whose ballistic equations are Table 1:

### 2.4. Gear Tooth Design

**Figure 4:**
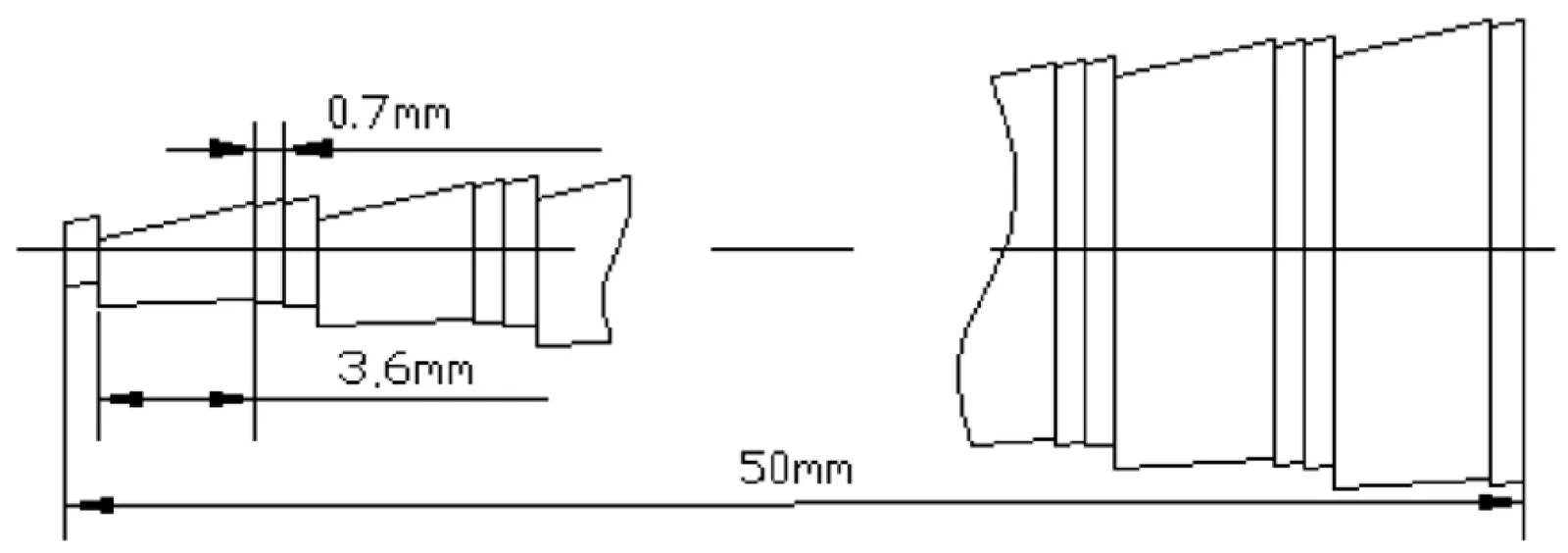
Dimension diagram.

When the cathode moves according to the variable-pitch helical line, the transmission mechanism inside the cathode body drives several working gears to move at different angular velocities. The total length of the working teeth composed of 30 wheel-shaped toothed disks is 50 mm. As shown in Figure 4, The thickness of each toothed disk ranges from 0.7 mm to 3.6 mm. When the cathode moves according to the variable-pitch helical equation, each tooth rotates at a different angle, and adjacent toothed disks are arranged in a staggered layout, enabling the overall inclination angle of the integrated working tooth to vary synchronously with the real-time helix angle of the processed variable-pitch helical line.

## 3. Bionic Design of Working Tooth Profile Surface

### 3.1. Biomimetic Drag Reduction Mechanism and Working Tooth Profile Design

**Figure 5:**
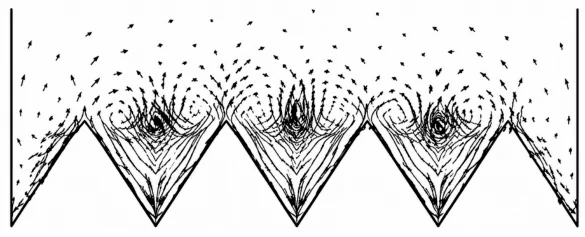
Drag reduction mechanism diagram.

**Figure 6:**
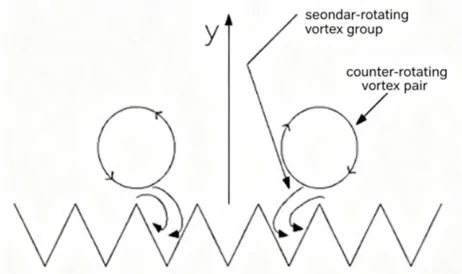
Interaction between electrolyte and sharp corners.

The drag reduction mechanism of biomimetic structures is illustrated in Figure 5. In the inter-electrode gap, the electrolyte flow rate is relatively high. When the turbulent electrolyte flows through the sharp corners of the cathode, it interacts with the corner structure to form secondary vortices, which isolate the flow vortices from the groove bottom. As shown in Figure 6, viscosity results in a low flow velocity near the groove bottom and high velocity near the top, which thickens the viscous sublayer accordingly. The fluid flows along the near-wall viscous sublayer rather than directly contacting the solid surface, which reduces the energy loss and the contact area between fluid and wall surface. Consequently, this reduces the shear stress within the boundary layer, leading to a reduced surface frictional drag. This achieves the drag reduction effect of the cathode biomimetic structure [9,10,11].

**Figure 7:**
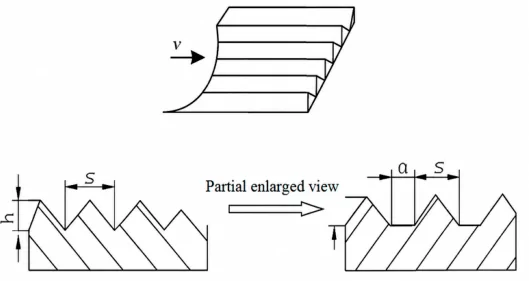
Biomimetic surface schematic.

During the electrochemical machining process, the electrolyte in the inter-electrode gap is in a turbulent state. By utilizing bionic drag reduction technology to control the turbulent structure within the turbulent boundary layer, the wall shape is altered to modify the flow field near the wall, thereby reducing the frictional resistance of the wall and achieving the goal of drag reduction [12,13]. In this optimization, the V-groove riblet biomimetic structure depicted in Figure 7 is employed to optimize the cathode working teeth. The primary focus is on investigating the impact of biomimetic structures of varying sizes and shapes on the drag reduction effect at a constant speed.

**Figure 8:**
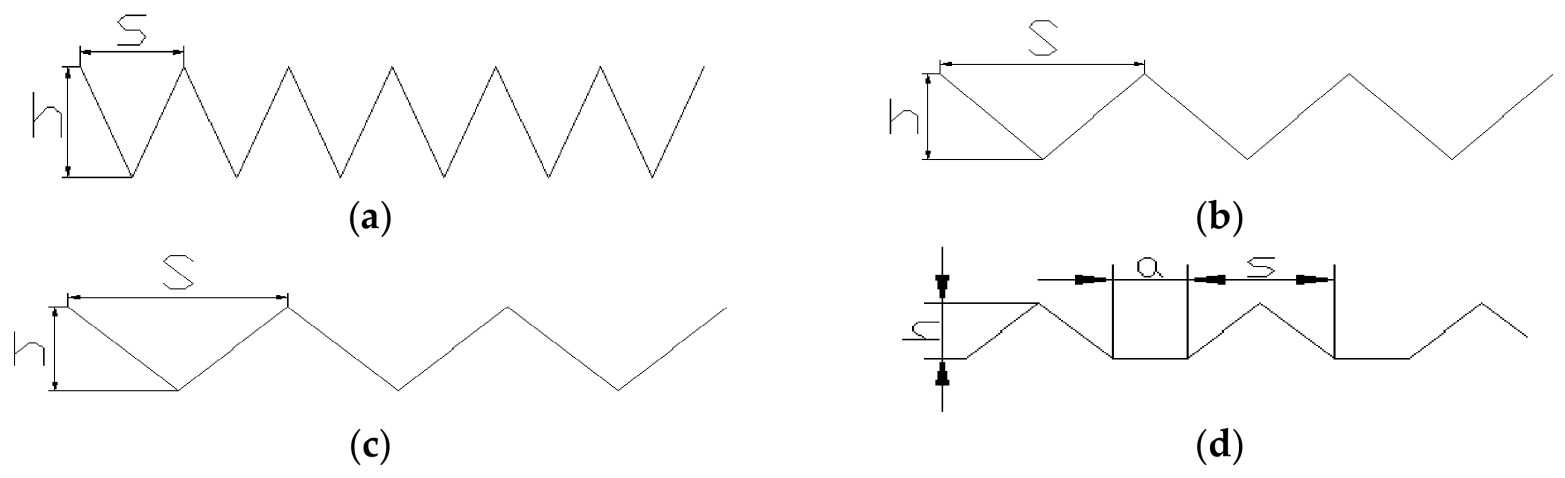
Different size shape structure diagram. (**a**) s = h = 0.1 mm; (**b**) h = 0.05 mm, s = 0.1 mm; (**c**) s = 0.2 mm, h = 0.05 mm; (**d**) s = h = a = 0.05 mm.

The current global research shows three optimal proportional forms of V-groove riblet biomimetic structures which have been verified to possess the optimal drag reduction performance: s/h = 1, s/h = 2, and s:h:a = 1 [14,15,16]. This paper conducts simulation calculations for the aforementioned three biomimetic structures, as well as for a biomimetic structure with s/h = 4. Given that the minimum width of the cathode working tooth is 0.7 mm, the bionic structural dimensions should be designed reasonably. Figure 8 presents schematic diagrams of four different sizes and shapes of biomimetic structures, with the basic dimension set as s/h = 1 (i.e., s = h = 0.05 mm, a = 0 mm, hereinafter abbreviated as s = h = 0.05 mm), and s/h = 2 (i.e., s = 0.1 mm, h = 0.05 mm, a = 0 mm, hereinafter abbreviated as s = 0.1 mm, h = 0.05 mm), as well as s = 0.2 mm, h = 0.05 mm, a = 0 mm (hereafter abbreviated as s = 0.2 mm, h = 0.05 mm) and s = h = a = 0.05 mm.

### 3.2. Optimization of Biomimetic Structures

To compare the four different size and shape structures shown in Figure 7, drag reduction calculations were performed. The shear stress (τ), which indicates the drag reduction effect, is calculated as follows [17,18,19]:

τ=μγ

where:

*τ*—shear stress;

*μ*—dynamic viscosity, Pa·s;

*γ*—shear rate.

To compare the frictional force and drag reduction performance of the four biomimetic structures with different structural parameters, a comparative analysis on electrolyte flow field parameters in the inter-electrode gap of electrochemical machining is carried out. COMSOL software 6.4 is employed to simulate the flow field in the inter-electrode gap, and the distribution clouds and distribution curves of its velocity, turbulence parameters, and shear rate are compared.

#### 3.2.1. Computer Simulation of Bionic Surface Velocity

(1) Inter-electrode gap model.

In electrochemical machining, increasing the electrolyte flow velocity can quickly take away electrochemical erosion sludge. Turbulent electrolyte flow can eliminate electrolyte concentration polarization, so the RNG k-ε turbulence model is adopted for simulation [20,21,22]:

$$\frac{\partial \left(\rho k\right)}{\partial t}+\frac{\partial \left(\rho ku_{i}\right)}{\partial x_{i}}=\frac{\partial }{\partial x_{j}}\left[\left(\mu +\frac{\mu_{i}}{\sigma_{k}}\right)\frac{\partial k}{\partial x_{j}}\right]+\rho_{k}-\rho \varepsilon$$

$$\frac{\partial \left(\rho \varepsilon \right)}{\partial t}+\frac{\partial \left(\rho \varepsilon u_{i}\right)}{\partial x_{i}}=\frac{\partial }{\partial x_{j}}\left[\left(\mu +\frac{\mu_{i}}{\sigma_{\varepsilon }}\right)\frac{\partial \varepsilon }{\partial x_{j}}\right]+\frac{C_{c1}}{k}\rho_{k}-C_{c2}\rho \frac{\varepsilon^{2}}{k}$$

In this equation, *k* denotes the turbulent kinetic energy, *ε* denotes the turbulent dissipation rate, *t* denotes time, *ρ* denotes the fluid density, $\mu_{i}$ denotes the turbulent viscosity, and $\rho_{k}$ the second term denotes the production term of k due to the mean velocity gradient

(2) Boundary conditions.

According to the definition formula of mass conservation, $\rho_{1}u_{1}A_{1}=\rho_{2}u_{2}A_{2}$

For a homogeneous and incompressible fluid, the formula becomes $u_{1}A_{1}=u_{2}A_{2}$

As shown in Table 2, *u*_1_ represents the inlet velocity of 15 m/s, *u*_2_ represents the outlet velocity, and A_1_ and A_2_ represent the inter-electrode gap areas at the inlet and outlet, respectively. The values of A_1_ and A_2_ measured by UG software 10.0 are 0.852415459 mm^2^ and 1.777412143 mm^2^, respectively. Therefore,

$$u_{2}=\frac{u_{1}A_{1}}{A_{2}}=\frac{15\times 0.852415459}{1.777412143}\approx 7.19373\;m/s$$

**Figure 9:**
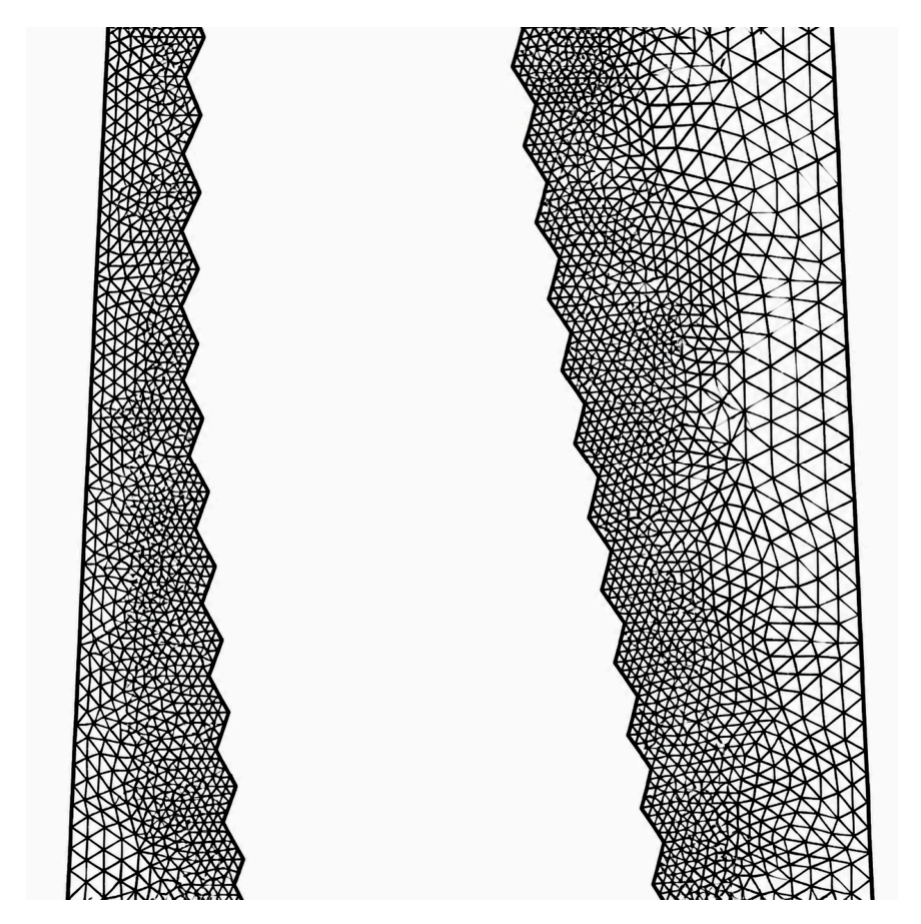
Meshing.

The calculated outlet velocity *u*_2_ is 7.19373 m/s under the set boundary conditions. In the ideal environment of COMSOL, during grid generation, it is also necessary to perform refinement on the sharp corners of the working tooth profile surface. The effect is shown in Figure 9.

**Figure 10:**
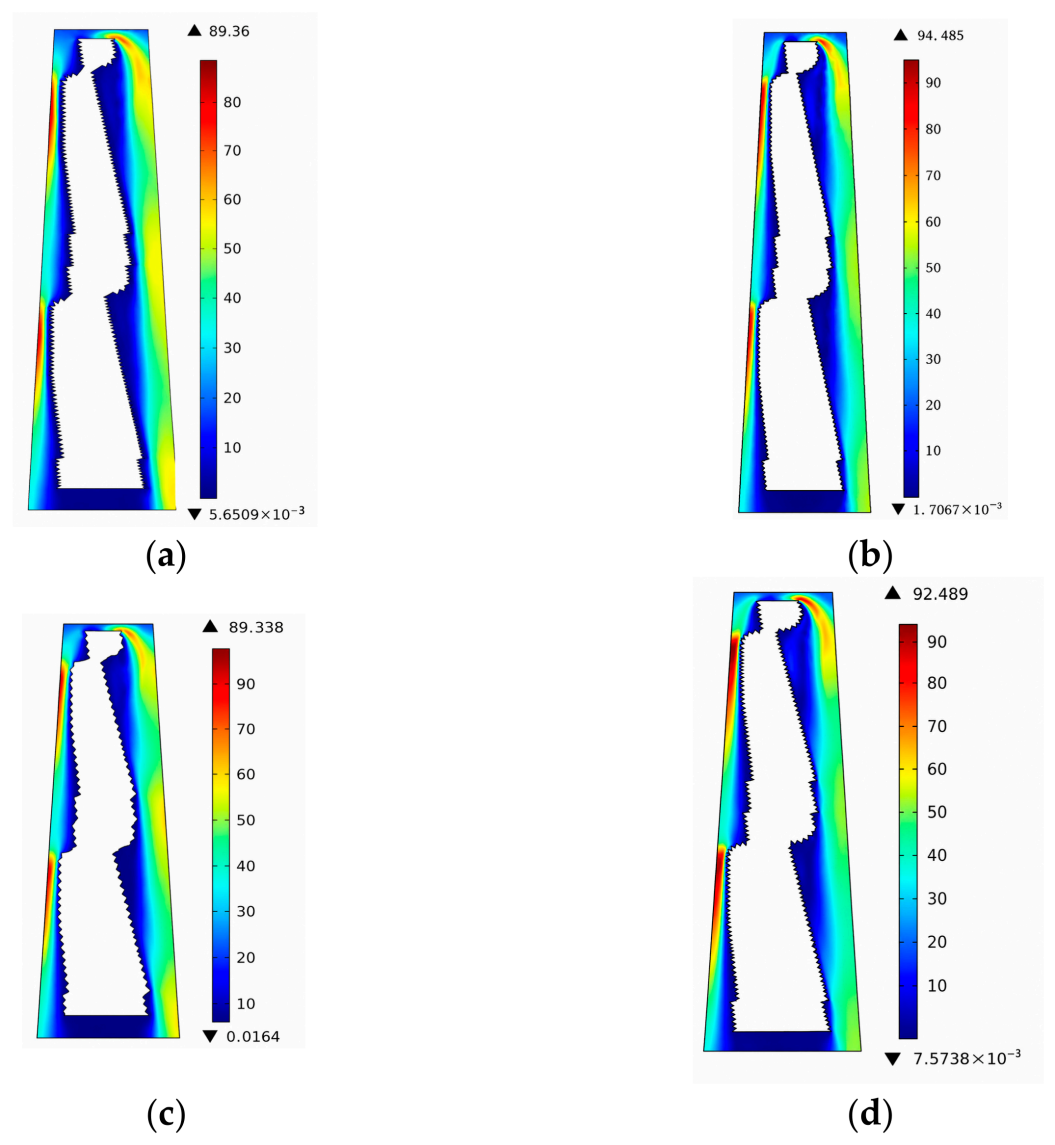
Velocity field distribution of biomimetic structure. (**a**) s = h = 0.1 mm; (**b**) s = 0.1 mm, h = 0.05 mm; (**c**) s = 0.2 mm, h = 0.05 mm; (**d**) s = h = a = 0.05 mm.

Figure 9 depicts the velocity distribution maps of four bionic surface sizes. All four biomimetic structures with different shapes and sizes have improved the distribution of the inter-electrode gap flow field. The velocity contour maps for the four shapes and sizes are basically consistent. Regardless of the shape and size, the electrolyte flow velocity near the biomimetic structure is stable and relatively slow, which also proves the existence of low-velocity zones in the viscous sublayer. The maximum velocity in Figure 10a reaches 89.36 m/s, while Figure 10b shows a maximum velocity of 94.485 m/s. Figure 10c presents the lowest peak velocity of 89.338 m/s, and Figure 10d shows a maximum velocity of 92.489 m/s. Upon a comprehensive comparison, Figure 10c has the lowest maximum velocity, corresponding to the most uniform velocity distribution and a relatively stable flow field.

**Figure 11:**
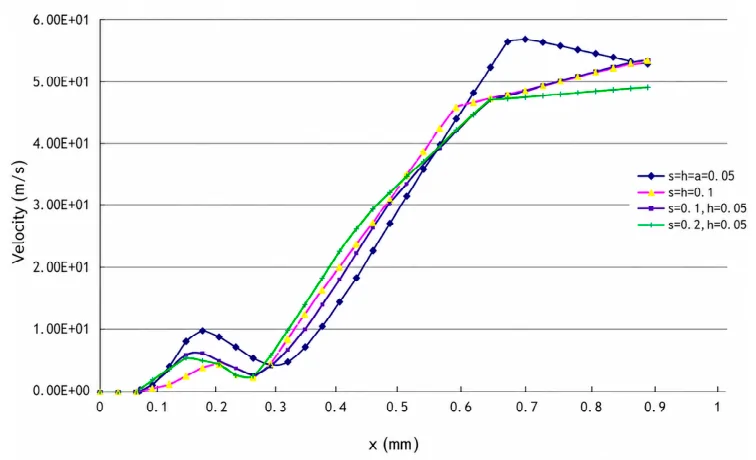
Velocity distribution curve.

Figure 11 depicts the velocity distribution curves for four different sizes of biomimetic structures, clearly showing that the velocity distribution trends for the bionic surfaces of these four sizes are essentially identical. The velocity presents a parabolic secondary distribution, which corresponds to the velocity distribution of a secondary vortex inside the biomimetic structure. The electrolyte forms a secondary vortex at the bottom of the biomimetic structure. Due to the influence of viscous drag, there is a low-velocity flow zone at the bionic bottom, leading to an increased thickness of the viscous sublayer and a reduced velocity gradient, which, in turn, reduces the shear stress and, subsequently, the friction resistance. When s = h = a = 0.05 mm, the parabolic distribution is the most obvious, with the vortex velocity reaching the maximum and exerting a significant inhibitory effect on the surrounding electrolyte, resulting in increased shear stress. The velocity curves for s = h = 0.1 mm and s = 0.1 mm, h = 0.05 mm almost coincide. When s = 0.2 mm and h = 0.05 mm, the velocity variation is relatively slow, with the smallest velocity gradient, leading to reduced shear stress.

#### 3.2.2. Turbulence Parameter Analysis

(1) Surface turbulent kinetic energy.

**Figure 12:**
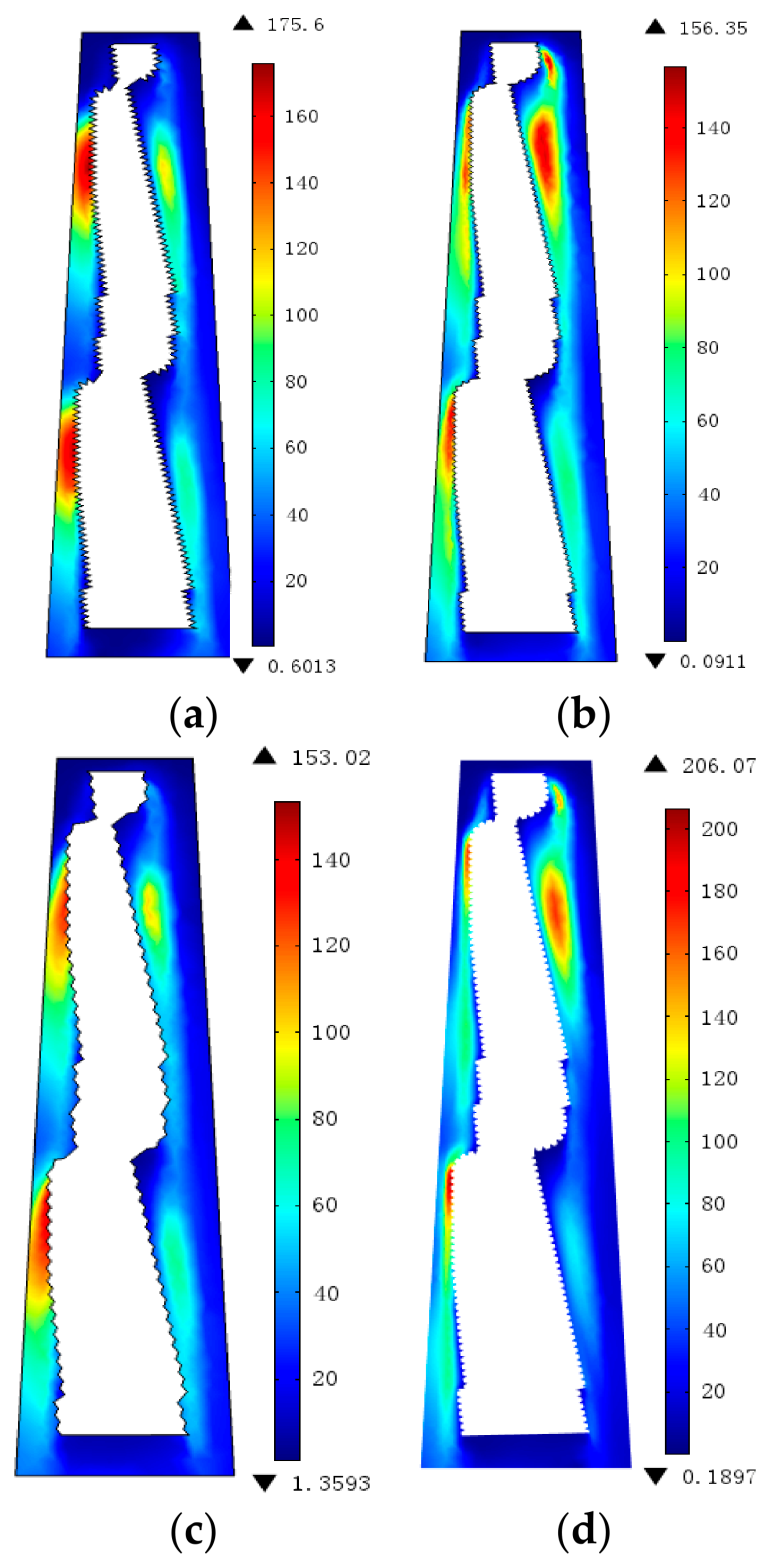
Cloud chart of turbulent kinetic energy distribution for four bionic models. (**a**) s = h = 0.1 mm; (**b**) s = 0.1 mm, h = 0.05 mm; (**c**) s = 0.2 mm, h = 0.05 mm; (**d**) s = h = a = 0.05 mm.

Through a comparison, it can be concluded that the turbulent kinetic energy reaches the minimum when the bionic structural parameters are s = 0.2 mm and h = 0.05 mm. The turbulent kinetic energy denoted by k is compared in terms of magnitude and distribution in the boundary layer of bionic surfaces with different sizes and shapes, as shown in Figure 12. A smaller turbulent kinetic energy means a lower energy consumption of the boundary layer, which can stabilize the boundary layer fluid significantly and reduce the flow resistance.

**Figure 13:**
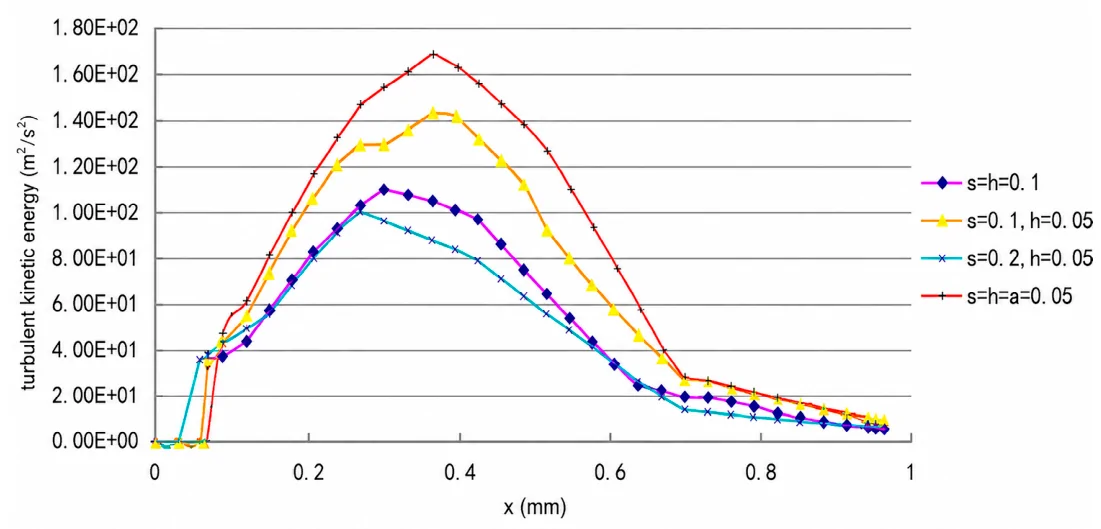
The change law of turbulent kinetic energy.

Figure 13 depicts the distribution curve of turbulent kinetic energy. By comparing the distribution curves, it can be observed that the change in turbulent kinetic energy on the biomimetic surface with s = 0.2 mm and h = 0.05 mm is relatively smooth. This smoothness reduces the turbulence fluctuations within the boundary layer, leading to a more stable flow field within the boundary layer, thereby achieving the goal of drag reduction.

(2) Turbulent kinetic energy dissipation rate.

**Figure 14:**
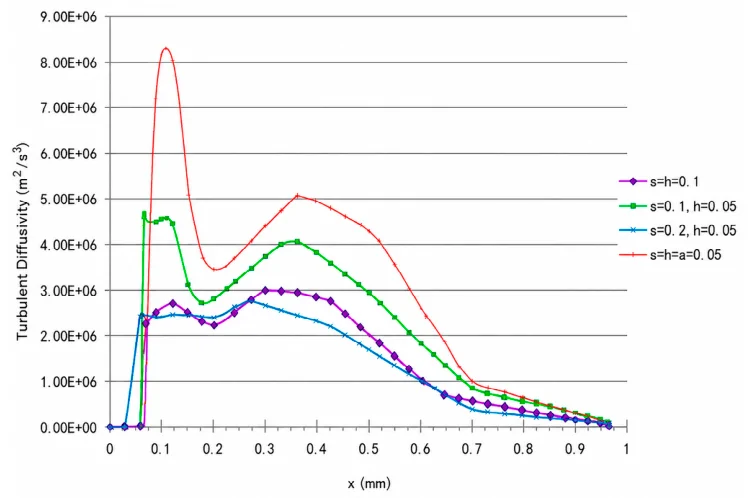
Dissipation rate curve.

Figure 14 depicts the distribution curve of the turbulence dissipation rate. It can be observed from the curve that, within the biomimetic structure and in the near-wall region on its surface, the low-speed secondary vortex within the biomimetic structure significantly suppresses turbulence. The turbulence dissipation rate of the bionic surface with s = 0.2 mm and h = 0.05 mm rises gently in the near-wall region, and the energy conversion in the boundary layer is not intense, resulting in a relatively stable flow field in the boundary layer.

#### 3.2.3. Shear Rate

**Figure 15:**
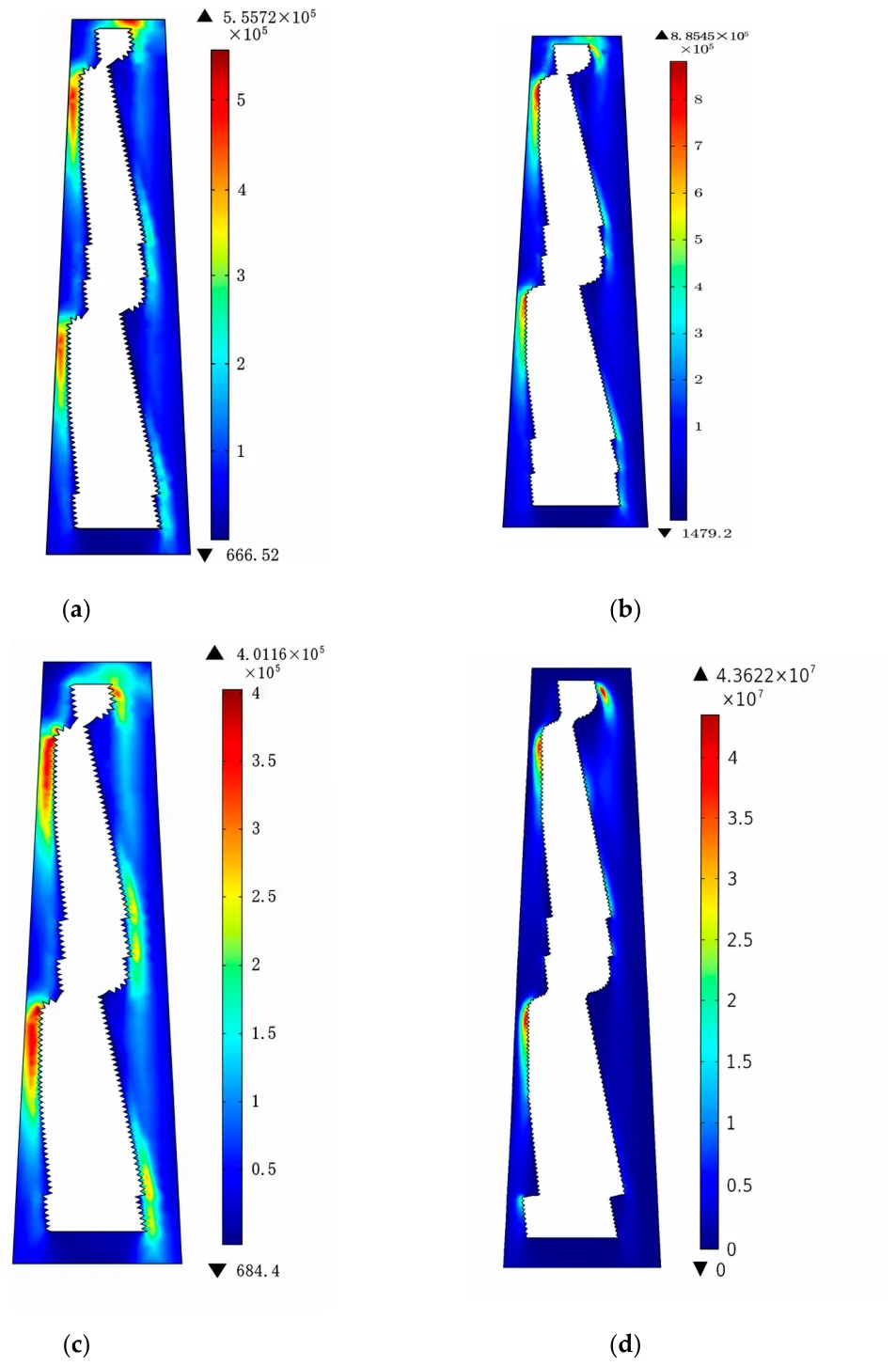
Shear rate distribution map. (**a**) s = h = 0.1 mm; (**b**) s = 0.1 mm, h = 0.05 mm; (**c**) s = 0.2 mm, h = 0.05 mm; (**d**) s = h = a = 0.05 mm.

**Figure 16:**
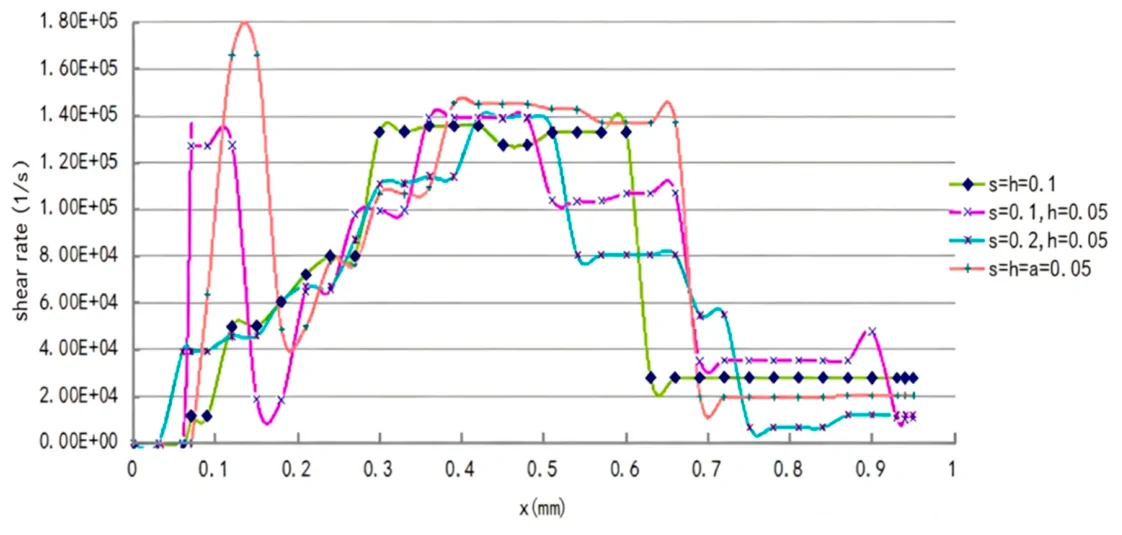
Shear rate distribution curve.

Figure 15 presents the comparative contour plots of the shear rate on the biomimetic surface, while Figure 16 shows the distribution curve of the shear rate. Under the constant dynamic viscosity of the fluid, the shear rate is positively correlated with the shear stress. The shear rate in Figure 15b is the highest at 8.8545 × 10^5^/s, while that in Figure 15c is the lowest at 4.0116 × 10^5^/s. From the curve of the shear rate variation on the biomimetic surface, it can be observed that the shear rate trends are roughly similar for the four sizes of biomimetic surfaces. At the bottom of the biomimetic structure, due to the influence of the secondary vortex, the fluid is in a viscous sublayer, and the shear stress reaches its minimum. Some fluid with a higher energy and velocity continues to move towards the top of the biomimetic structure, but the movement of the secondary vortex hinders the progress of this fluid. Upon reaching the top of the biomimetic structure, the shear rate rapidly increases, and, at this point, some fluid has essentially exhausted its energy, causing a sharp decrease in the shear stress. When the biomimetic size is s = 0.2 mm and h = 0.05 mm, its shear stress is the smallest, indicating that its drag reduction effect is the best.

## 4. Experimental Verification

**Figure 17:**
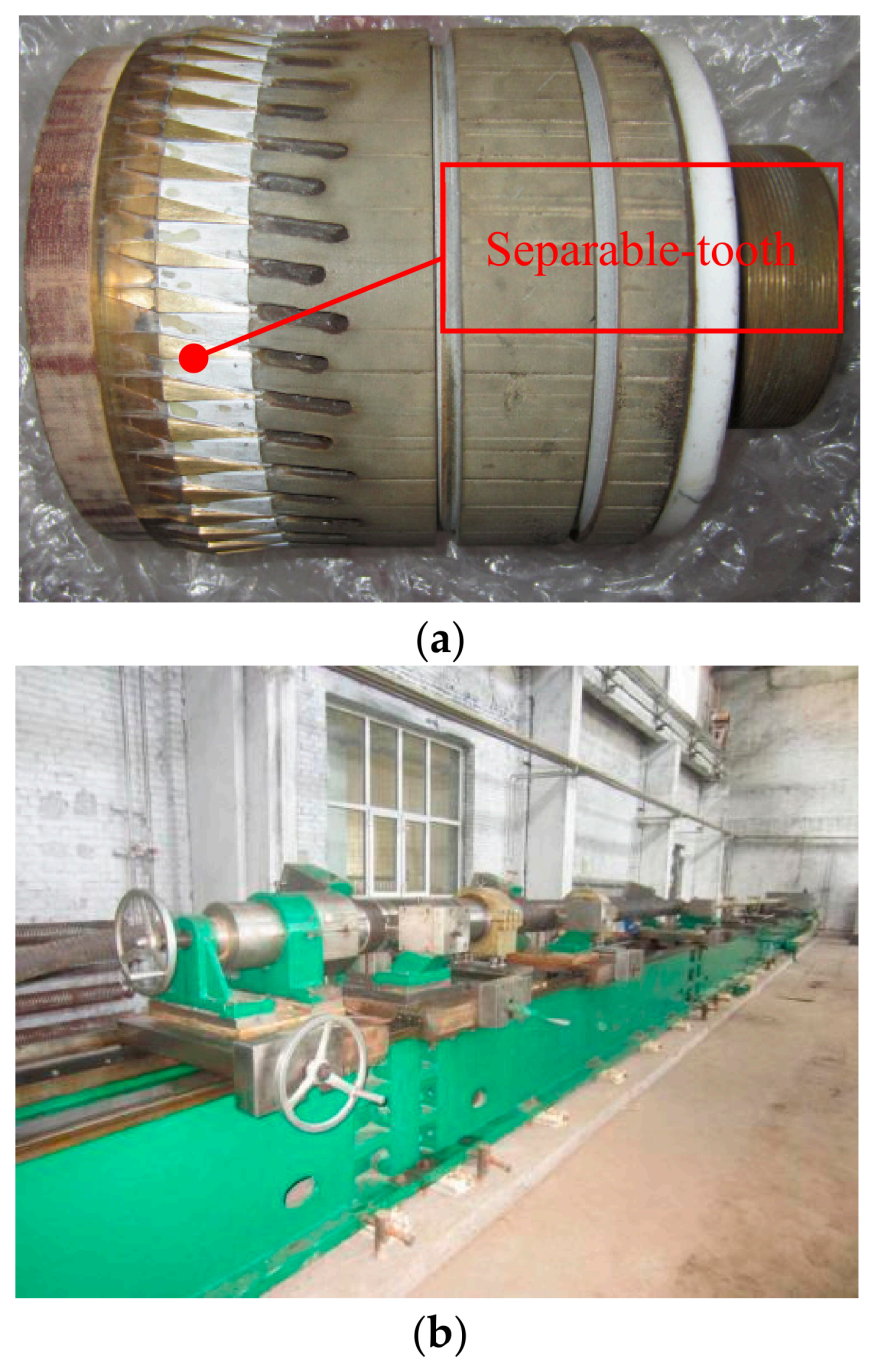
Separable-tooth cathode and processing site. (**a**) Separable-tooth cathode; (**b**) Processing site.

As shown in Figure 17, a cathode test was conducted on a large horizontal electrochemical machine tool, with the following electrochemical machining parameter settings: machining voltage: 10–13 V; feed rate: 12–16 mm/min; electrolyte: 15% NaCl; electrolyte pressure: 2 MPa; and electrolyte temperature: 35 °C. Process experiments were performed to verify the new cathode structural design. When the cathode travel reached 300 mm, the machining current rose to approximately 4300 A. The overall electrolytic machining condition remained stable without obvious current oscillation. Table 3, Table 4 and Table 5 presents some of the experimental data recorded.

**Figure 18:**
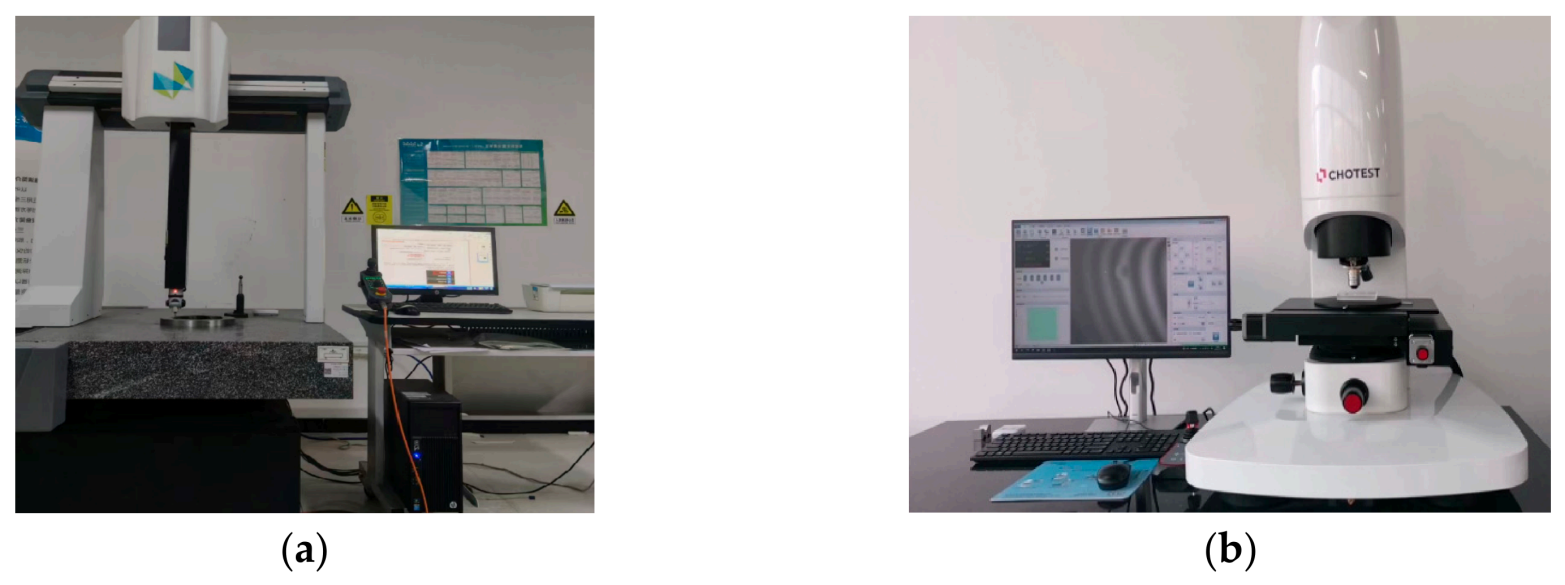
Measuring equipment. (**a**) Coordinate measuring machine; (**b**) 3D optical profilometer.

As shown in Figure 18, workpiece inspection was performed using a coordinate measuring machine, 3D optical profilometer, and dial indicator, among others. Specifically, the coordinate measuring machine was employed to acquire the part’s contour data, the 3D optical profilometer was used to characterize the three-dimensional microtopography and three-dimensional surface roughness of the workpiece, and the micrometer was utilized to assess the dimensional consistency of the workpiece.

**Figure 19:**
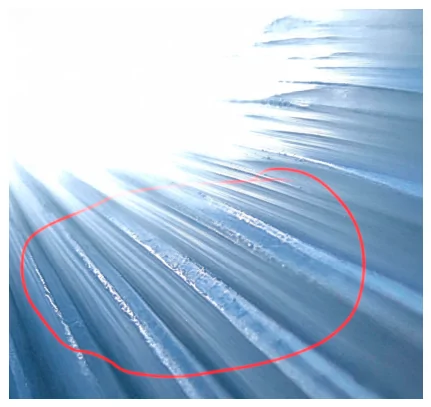
Ridge-like defect.

**Figure 20:**
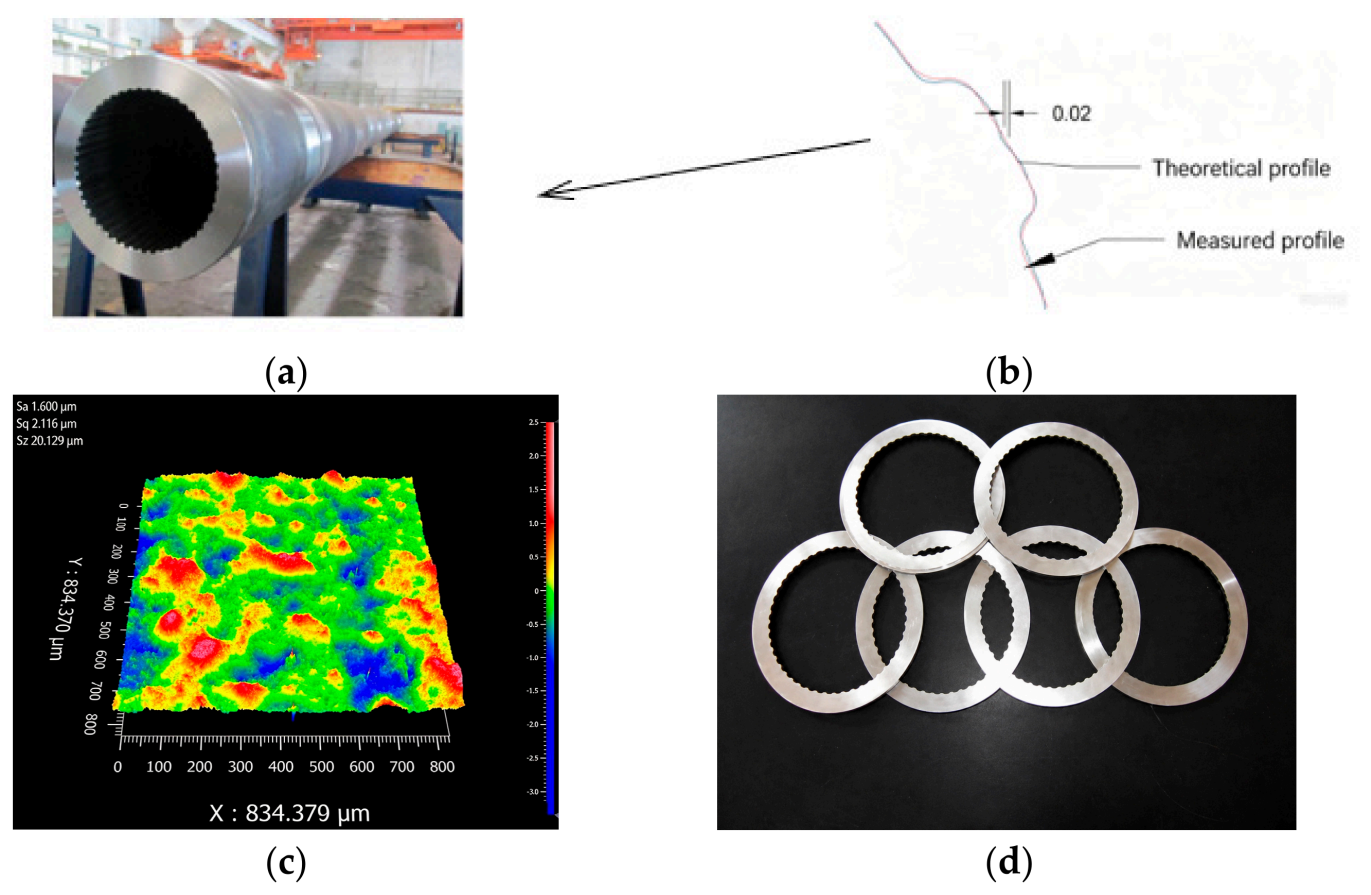
Parts and slices. (**a**) Part; (**b**) Forming precision; (**c**) Surface roughness; (**d**) Slice.

Figure 19 shows the inner hole surface of a workpiece processed using conventional cathode electrolytic machining. Based on the morphological characteristics, it can be clearly observed that the positive contour of the inner hole in conventional cathode machining exhibits irregularities, with prominent missing corners and contour defects, resulting in a poor forming consistency and surface quality. Figure 20 presents the machining results achieved using the novel bionic segmented cathode. Compared to the conventional cathode structure, the machining performance of the new bionic cathode has been significantly optimized, achieving a dimensional accuracy of the workpiece inner hole of ±0.02 mm and a surface roughness Sa 1.6 μm. Experimental results demonstrate that the use of the bionic segmented cathode effectively suppresses machining defects such as missing corners and contour distortion in inner holes, substantially enhances the forming precision and surface quality of the inner holes, and ensures a stable overall machining process with a high process reliability.

## 5. Results and Discussion

### 5.1. Design and Analysis of Split-Type Cathode Working Teeth

In this paper, a novel bionic cathode structure is proposed. The working teeth of the cathode are discretized into a set of gear-shaped working discs with distinct reference circle diameters. During the machining process, the cathode moves according to the variable-pitch helix motion equation, while the gear-shaped working discs are driven by the embedded micro-motor and transmission mechanism to rotate relative to the cathode body. Benefiting from the difference in reference circle diameters, each working disc produces a unique rotation angle. This configuration enables the envelope curve of the integral working teeth to consistently align with the helix angle of the machined helix, thereby effectively preventing the thread wall collapse defect of the machined profile.

### 5.2. Optimization of Bionic Structural Parameters

A comparative analysis of turbulent flow parameters is conducted to identify the optimal bionic geometry. A quantitative evaluation of the shear stress, velocity gradient, turbulent kinetic energy, and flow field uniformity indicates that the bionic structure with spacing s = 0.2 mm and height h = 0.05 mm delivers the best drag reduction performance. The surface shear stress is remarkably reduced, while the bottom shear stress remains sufficiently low. The optimized structure stabilizes the inter-electrode flow field, reduces the overall system resistance, and improves the flow uniformity and machining stability.

### 5.3. Experimental Verification and Prospect

Extensive ECM trials are performed to verify the numerical results and engineering feasibility. The sectioned measurement and surface characterization demonstrate that the inner hole achieves surface roughness Sa1.6 μm, roundness 0.02 mm, and dimensional consistency within 0.02 mm, all of which satisfy the high-precision manufacturing requirements. The experimental results confirm that the split-type cathode generates a uniform and stable inter-electrode flow field, effectively eliminating local burning, collapse, and profile widening. Therefore, through the innovative structural design, numerical simulation, and experimental investigation of the separable-tooth cathode for the electrochemical machining of large-diameter barrels, this study broadens the technical system for the electrochemical machining of complex variable-pitch helical structures. However, electrochemical machining is affected by numerous coupled factors. To further improve the machining accuracy of workpieces, further systematic explorations and investigations are still needed in future research.

## Figures and Tables

**Table 1:** Helix geometry.

Segment	Ballistic Equation Y(X)	tan α = dY/dX	Helix Angle	Range
Starting uniform	0.034921 X	0.034921	2°	X ≤ 150
Gain twist	1.0628(X + 47.0396)^1.575^/1000 + 0.86942	1.67391 × 10^−3^ · (X + 47.0396)^0.575^	2° → 7.5°	150 < X ≤ 1934
Muzzle uniform	13.1653(X − 1934)/100 + 166.46845	0.131653	7.5°	X > 1934

**Table 2:** Boundary conditions and electrolyte properties.

Item	Setting
Inlet boundary	velocity inlet, *u*_1_ = 15 m/s
Outlet boundary	pressure outlet
Turbulence model	RNG k–ε
Turbulence intensity	5–10%
Hydraulic diameter	LT = 0.07·Dh
Electrolyte	Nacl
Density, viscosity, conductivity	measured values at the test temperature
Temperature dependence	κ = κ0[1 + ξ(T − T0)]
Mesh	locally refined at tooth tips and structure edges

**Table 3:** First experiment.

Serial No.	Groove Width (mm)	Groove Diameter (mm)
1	6.27	159.66
2	6.26	159.68
3	6.25	159.64
4	6.25	159.64
5	6.27	159.68
6	6.25	159.64
7	6.26	159.64
8	6.26	159.66
9	6.27	159.66
10	6.26	159.64
11	6.25	159.64
12	6.26	159.64
13	6.27	159.66
14	6.26	159.76
15	6.26	159.68
16	6.26	159.68
17	6.27	159.66

**Table 4:** Second experiment.

Serial No.	Groove Width (mm)	Groove Diameter (mm)
1	6.26	159.68
2	6.26	159.66
3	6.26	159.66
4	6.26	159.68
5	6.28	159.68
6	6.28	159.64
7	6.28	159.66
8	6.24	159.66
9	6.26	159.66
10	6.26	159.66
11	6.28	159.66
12	6.26	159.68
13	6.28	159.68
14	6.28	159.68
15	6.26	159.68
16	6.26	159.68
17	6.28	159.68

**Table 5:** Three experiment.

Serial No.	Groove Width (mm)	Groove Diameter (mm)
1	6.26	159.64
2	6.26	159.66
3	6.26	159.68
4	6.28	159.66
5	6.26	159.64
6	6.26	159.64
7	6.28	159.64
8	6.26	159.68
9	6.26	159.66
10	6.24	159.64
11	6.24	159.68
12	6.26	159.66
13	6.28	159.64
14	6.26	159.64
15	6.24	159.66
16	6.24	159.68
17	6.26	159.66

## Data Availability

The original contributions presented in this study are included in the article. Further inquiries can be directed to the corresponding author.
